# Identification of PP2A and S6 Kinase as Modifiers of Leucine-Rich Repeat Kinase-Induced Neurotoxicity

**DOI:** 10.1007/s12017-019-08577-z

**Published:** 2019-10-29

**Authors:** Joan Poh Ling Sim, Wang Ziyin, Adeline Henry Basil, Shuping Lin, Zhongcan Chen, Chengwu Zhang, Li Zeng, Yu Cai, Kah-Leong Lim

**Affiliations:** 1 Neurodegeneration Research Laboratory, National Neuroscience Institute, 11, Jalan Tan Tock Seng, Singapore, 308433 Singapore; 2 Neural Stem Cell Research Laboratory, National Neuroscience Institute, Singapore, Singapore; 3 Nanjing Tech University, Nanjing, China; 4 Temasek Life Science Laboratories, Singapore, Singapore; 5 Department of Physiology, National University of Singapore, Singapore, Singapore; 6 Duke-NUS Medical School, Singapore, Singapore

**Keywords:** Parkinson’s disease, LRRK2, Mitochondria, Drosophila, Neuroprotection

## Abstract

Mutations in *LRRK2* are currently recognized as the most common monogenetic cause of Parkinsonism. The elevation of kinase activity of LRRK2 that frequently accompanies its mutations is widely thought to contribute to its toxicity. Accordingly, many groups have developed LRRK2-specific kinase inhibitors as a potential therapeutic strategy. Given that protein phosphorylation is a reversible event, we sought to elucidate the phosphatase(s) that can reverse LRRK2-mediated phosphorylation, with the view that targeting this phosphatase(s) may similarly be beneficial. Using an unbiased RNAi phosphatase screen conducted in a *Drosophila* LRRK2 model, we identified PP2A as a genetic modulator of LRRK2-induced neurotoxicity. Further, we also identified ribosomal S6 kinase (S6K), a target of PP2A, as a novel regulator of LRRK2 function. Finally, we showed that modulation of PP2A or S6K activities ameliorates LRRK2-associated disease phenotype in *Drosophila*.

**Electronic supplementary material:**

The online version of this article (10.1007/s12017-019-08577-z) contains supplementary material, which is available to authorized users.

## Introduction

Parkinson’s disease (PD) is a prevalent neurodegenerative movement disorder characterized pathologically by the rather selective loss of dopaminergic (DA) neurons in the substantia nigra pars compacta (SNpc). Although most cases of PD occur in a sporadic manner, a subset of PD cases is inheritable and attributable to mutations in specific genes (Chai and Lim 2013). Among these, mutations in the *Leucine*-*rich repeat kinase 2* (*LRRK2*) gene are currently recognized as the most prevalent monogenetic cause of Parkinsonism (Paisan-Ruiz et al. 2004; Zimprich et al. 2004). To date, a large number of LRRK2 mutations have been identified, with the G2019S variant being the most common. In general, disease-associated mutations of LRRK2 tend to increase its kinase activity and thereby its toxicity (Martin et al. 2014a). Given this, modulating LRRK2 kinase function represents an intuitive therapeutic focus and several LRRK2 inhibitors have been developed that have potential disease-modifying properties (Taymans and Greggio 2016). As protein phosphorylation is a reversible event, an alternative approach is to elucidate the phosphatase(s) that can reverse LRRK2-mediated phosphorylation, with the view that the activation of this phosphatase via pharmacological or genetic means would work in a similarly beneficial fashion. This is the approach that we have taken here. Using our previously described *Drosophila* LRRK2 G2019S mutant (Ng et al. 2009) as a model, we conducted an unbiased RNAi phosphatase screen and identified Protein Phosphatase 2A (PP2A) as a genetic modifier of LRRK2-induced neurotoxicity. We further found that ribosomal S6 kinase (S6K), a recently identified target of PP2A (Hahn et al. 2010), exhibits enhanced phosphorylation in the presence of LRRK2, which suggests a functional relationship between the two proteins. Finally, we demonstrated that pharmacological or genetic activation of PP2A activity, or inhibition of S6K activity, mitigates LRRK2-associated disease phenotypes in *Drosophila*.

## Methods

### Fly Stocks

The list of phosphatase RNAi lines (Table S1) was provided by Dr. Cai Yu (Temasek Life Sciences Laboratory, Singapore). Other fly lines include 24B>UAS-LRRK2 G2019S, Ddc>UAS-LRRK2 G2019S, UAS-mts, UAS-wrd (kind gifts from Aurelio Teleman, German Cancer Research Center, Germany), UAS-wrd RNAi (Vienna Drosophila Stock Center, Austria), UAS-S6K RNAi (VDRC) and UAS-S6K KQ (kind gifts from Mary Stewart, North Dakota State University, USA). All the fly lines used in our assays are in the same genetic background i.e. genotype *yw* (yellow-white). In general, control flies refer to the native *yw* line.

### Climbing Assay and Drug Treatment

Climbing assays were carried out according to previously described methods (Ng et al. 2009). In general, 30 flies per group were used for the assay and the experiment was replicated with three different sets of flies. To study the effects of drugs, flies were fed with cornmeal-agar medium supplemented with 250 μM C2 Ceramide (*N*-acetyl-d-sphingosine, Sigma-Aldrich), 250 μM Fingolimod (FTY720), HCl (Selleckchem) or 250 μM S6K1 Inhibitor (PF-4708671, Tocris) at day 25 post-eclosion till day 50.

### Immunohistochemistry

Immunohistochemical analysis of whole-mount adult fly brains was performed according to published protocols (Whitworth et al. 2005) and stained with rabbit anti-TH (1:300, Pel-Freez Biologicals, Milwaukee) as primary antibody before analysis using an Olympus Fluoview Upright Confocal Microscope. DA neurons were quantified according to published methods (Whitworth et al. 2005). For each genotype, about 40–50 fly brains were analyzed. The size of mito-GFP puncta was measured using Image J program and expressed as mean ± S.E.M. (*n* ≥ 10 DA neurons per experimental group).

### Cell Culture and Western Blot Analysis

SH-SY5Y cells at 60–70% confluency were transfected with the indicated plasmids using Lipofectamine reagent (Thermo Fisher Scientific) as per manufacturer’s instructions. Cells were harvested 48-h post transfection by washing in cold PBS before sonication in RIPA lysis buffer. The lysate was collected after centrifugation at 13,000 rpm, 15 min, 4 °C. 40 μg of protein for each sample was used for electrophoresis (120 V) on 8% SDS-PAGE followed by wet transfer onto nitrocellulose membrane for 80 Volts, 3 h. The primary antibodies used are 1:1000 rabbit anti-PP2A-B (Cell-Signaling Technology, CST), 1:1000 rabbit anti-PP2A-C (CST), 1:2000 guinea pig anti-dS6K (generous gift from Aurelio Teleman), 1:1000 rabbit anti-LRRK2 (Sigma), 1:1000 rabbit anti-phospho-LRRK2 (Ser935) (Abcam), 1:1000 rabbit anti-phospho-LRRK2 (Ser1292) (Abcam), 1:1000 rabbit anti-S6K (CST), 1:1000 mouse anti-phospho-S6K (Thr389) (CST), 1:1000 rabbit anti-mTOR (CST), 1:1000 rabbit anti-phospho-mTOR (Ser2448) (CST) and 1:10,000 mouse anti-actin (Sigma). Detection was performed via chemiluminescence on a Kodak X-ray film developer.

### Primary Mouse Neurons

For primary cortical neuron culture, embryonic day 17.5 mouse fetuses from C57BL/6 and LRRK2 G2019S transgenic mice were obtained, and their meninges were removed. The cortex was isolated and dissociated with 0.25% trypsin for 20 min, followed by the addition of trypsin inhibitor. Cells were washed, titrated, and resuspended in neurobasal medium supplemented with B27 and GlutaMAX (Gibco). Mouse-related work was approved by and conformed to the guidelines of the TTSH-NNI Institutional Animal Care and Use Committee.

### Statistical Analysis

Unless otherwise stated, statistical significance for all the quantitative data obtained were analyzed using either unpaired Student’s *t* test (*p < 0.05, **p < 0.01, ***p < 0.001) or one-way ANOVA (*P < 0.05, **p < 0.01).

## Results

### RNAi-Based Phosphatase Screen Identified PP2A as a Candidate Genetic Modifier of LRRK2-Induced Toxicity

**Fig. 1:**
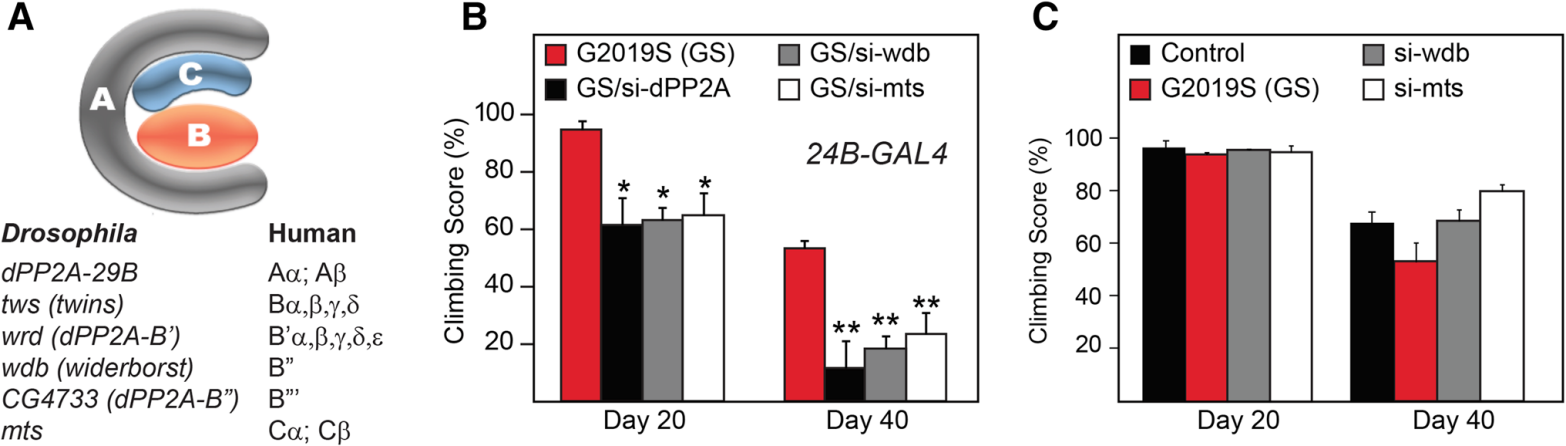
RNA-mediated knockdown of the expression of PP2A subunits aggravates the climbing deficits of transgenic LRRK2 G2019S flies. **a** Cartoon depicting the different PP2A subunits in human and flies. **b** Climbing score of *24B*-Gal4 driven LRRK2 G2019S transgenic flies at Day 20 and Day 40 post-eclosion in the absence or presence of siRNA-mediated silencing of expression of the various PP2A subunits, as indicated. **c** Climbing score of control (yw) and *24B*-Gal4 driven LRRK2 G2019S, siRNA-mediated silencing of wdb (dPP2A-B”) and mts (dPP2A-C) at Day 20 and Day 40 post-eclosion. *p < 0.05, **p < 0.01

Given that the elevation of kinase activity of LRRK2 that frequently accompanies its mutations is widely thought to underlie its toxicity, we sought to elucidate the cognate phosphatase(s) that can potentially reverse LRRK2-mediated phosphorylation. For this purpose, we conducted a RNAi-based phosphatase screen in the *Drosophila* LRRK2 G2019S mutant. Our expectation is that reduced expression of LRRK2-related phosphatase would aggravate its phenotype. We have previously demonstrated that the expression of LRRK2 G2019S mutant in the flight muscles of *Drosophila* (via the *24B*-GAL4 driver) results in significant impairments in their climbing ability (Ng et al. 2012), which provides a convenient readout. Altogether, we screened 39 phosphatases and identified 7 of them whose reduced expression worsens the climbing phenotype of LRRK2 mutant flies (Table S1). Among these, three are linked to Protein Phosphatase 2A, i.e. *dPP2A*-*29B*; *wdb (widerborst)* and *mts (microtubule star)*, whose products, respectively, represent the scaffold (A), regulatory (B) and catalytic (C) subunits of the holoenzyme (Fig. 1a). The reduced expression of these fly PP2A subunits via RNAi technology in the flight muscles of *Drosophila* LRRK2 G2019S mutant significantly retarded their locomotion ability and, in an age-dependent progressive manner (Fig. 1b), suggesting that PP2A is a potential genetic modifier of LRRK2-induced toxicity. Notably, these RNAi/UAS-PP2A subunit lines on their own did not trigger overt-climbing defects when compared to control flies (Fig. 1c).

### Genetic or Pharmacological Activation of PP2A Counteracts LRRK2-Induced Neurotoxicity

**Fig. 2:**
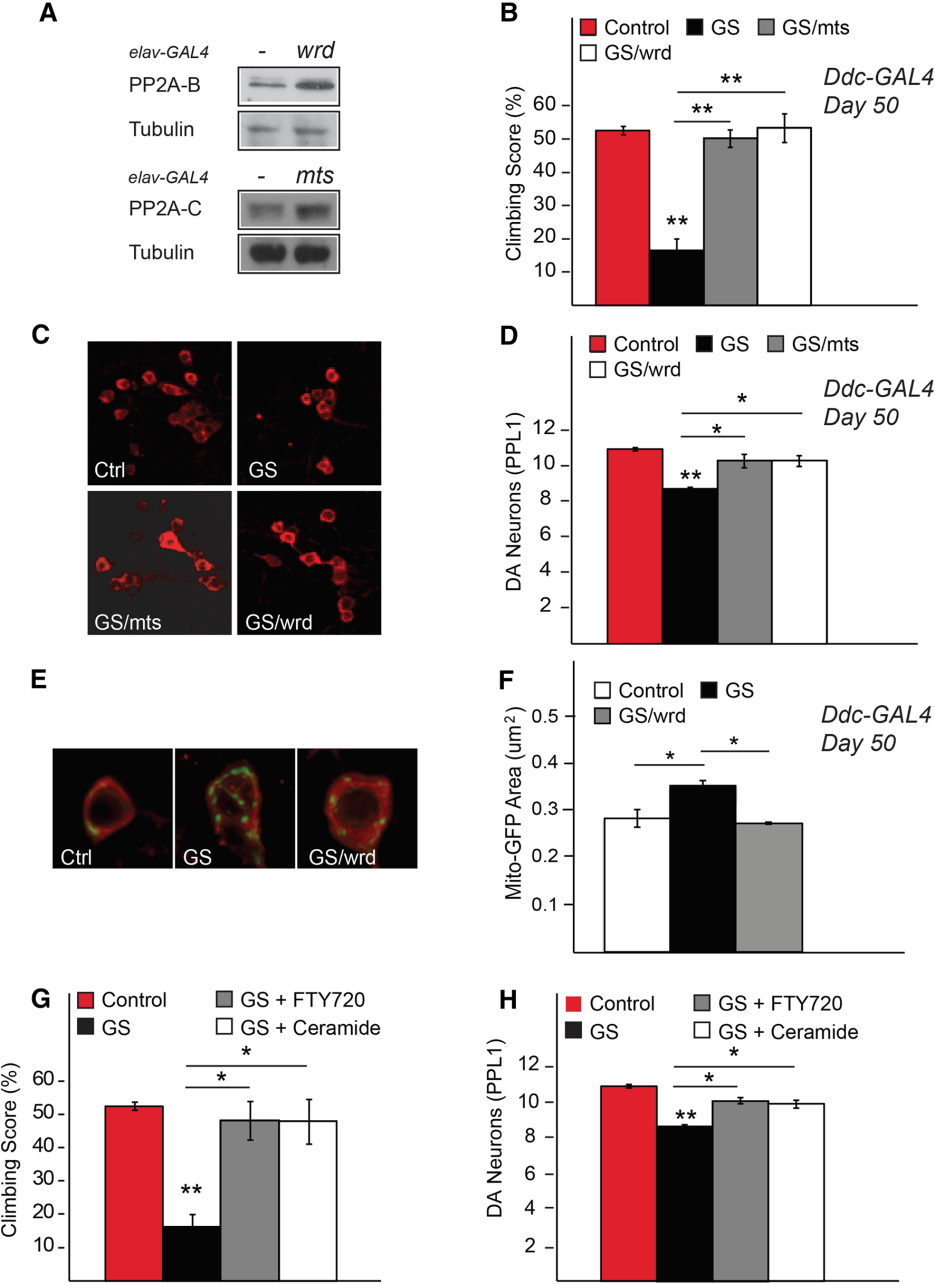
Expression of *Drosophila* PP2A subunits rescues the pathological phenotypes in transgenic LRRK2 G2019S flies. **a** Immunoblots showing the expression of wrd (dPP2A-B’) and mts (dPP2A-C) expression driven by the pan-neuronal *elav* driver. **b** Climbing score of *Ddc*-Gal4 driven LRRK2 G2019S transgenic flies (50 days post-eclosion) in the absence or presence of wrd or mts co-expression. **c** Representative confocal microscopy images showing TH-positive (red) dopaminergic neurons in the PPL1 cluster of control flies or those expressing LRRK2 G2019S mutant in the absence or presence of wrd or mts co-expression, as indicated. The accompanying bar-graph showing the number of TH-positive DA neurons in these flies is shown in **d**. **e** Representative images of mito-GFP (green) immunostaining in the cell bodies (red) of DA neurons of control flies or those expressing LRRK2 G2019S mutant in the presence or absence of wrd co-expression, as indicated. **f** Bar-graph showing the average size + S.E.M of mito-GFP puncta in control and mutant LRRK2 flies (G2019S) in the absence or presence of wrd co-expression. **g** Climbing score and **h** DA neuronal count of *Ddc*-Gal4 driven LRRK2 G2019S transgenic flies (50 days post-eclosion) in the absence or presence of FTY720 or Ceramide treatment, as indicated. *p < 0.05, **p < 0.01

Next, we sought to address whether the modulation of PP2A activity may influence the function of DA neurons harbouring LRRK2 mutations. For this purpose, we first ascertained the expression of *wrd (dPP2A*-*B’)* or *mts* in the fly brain driven by the pan-neuronal elav-GAL4 driver (Fig. 2a) and that their co-expression with LRRK2 did not affect the levels of LRRK2 expression (Fig. S1A). When these PP2A subunits are co-expressed with LRRK2 G2019S via the Ddc-GAL4 driver (which expresses in DA neurons), they provide significant protection against the loss of DA neurons in the PPL1 DA cluster in LRRK2 mutant flies that is accompanied by a marked improvement in their climbing ability (Fig. 2b–d). In general, we looked at the PPL1 cluster as LRRK2 G2019S expression does not appear to affect other DA clusters (not shown), and we carried out our rescue assay with LRRK2 mutant flies at day 50 post-eclosion as this is the time point where they exhibit the most apparent and significant climbing deficit compared to their control counterparts (Fig. S1B). Accompanying this rescue is the restoration of the neuronal mitochondrial phenotype in the presence of PP2A co-expression, which is otherwise abnormally enlarged when LRRK2 G2019S is expressed alone (Fig. 2e–f), as previously reported by our group (Ng et al. 2012).

Following this, we examined whether pharmacological activation of PP2A may likewise be beneficial to LRRK2-expressing flies. For this purpose, we treated LRRK2 G2019S-expressing flies with two well-documented PP2A activators, i.e. ceramide and fingolimod (FTY720) (Perrotti and Neviani 2013). Similar to genetic overexpression of fly PP2A subunits, ceramide- or fingolimod-treated mutant LRRK2 flies exhibit marked improvement in their DA neuronal count and their climbing ability (Fig. 2g, h). These PP2A-activating compounds otherwise have no effect on the climbing performance of treated control flies (Fig. S1C). Taken together, these results demonstrate that the modulation of PP2A activity via genetic or pharmacological means can counteract LRRK2-induced neurotoxicity, suggesting a protective mechanism that presumably involves the reversal of LRRK2-mediated phosphorylation by PP2A. Supporting this, we found that the autophosphorylation level of LRRK2 G2019S (Ser-1292) is reduced in the presence of PP2A-B co-expression (Fig. S1D). The kinase-dead LRRK2 D1994A was used as a negative control.

### Ribosomal S6 Kinase Exhibits Enhanced Phosphorylation in the Presence of LRRK2 Overexpression

**Fig. 3:**
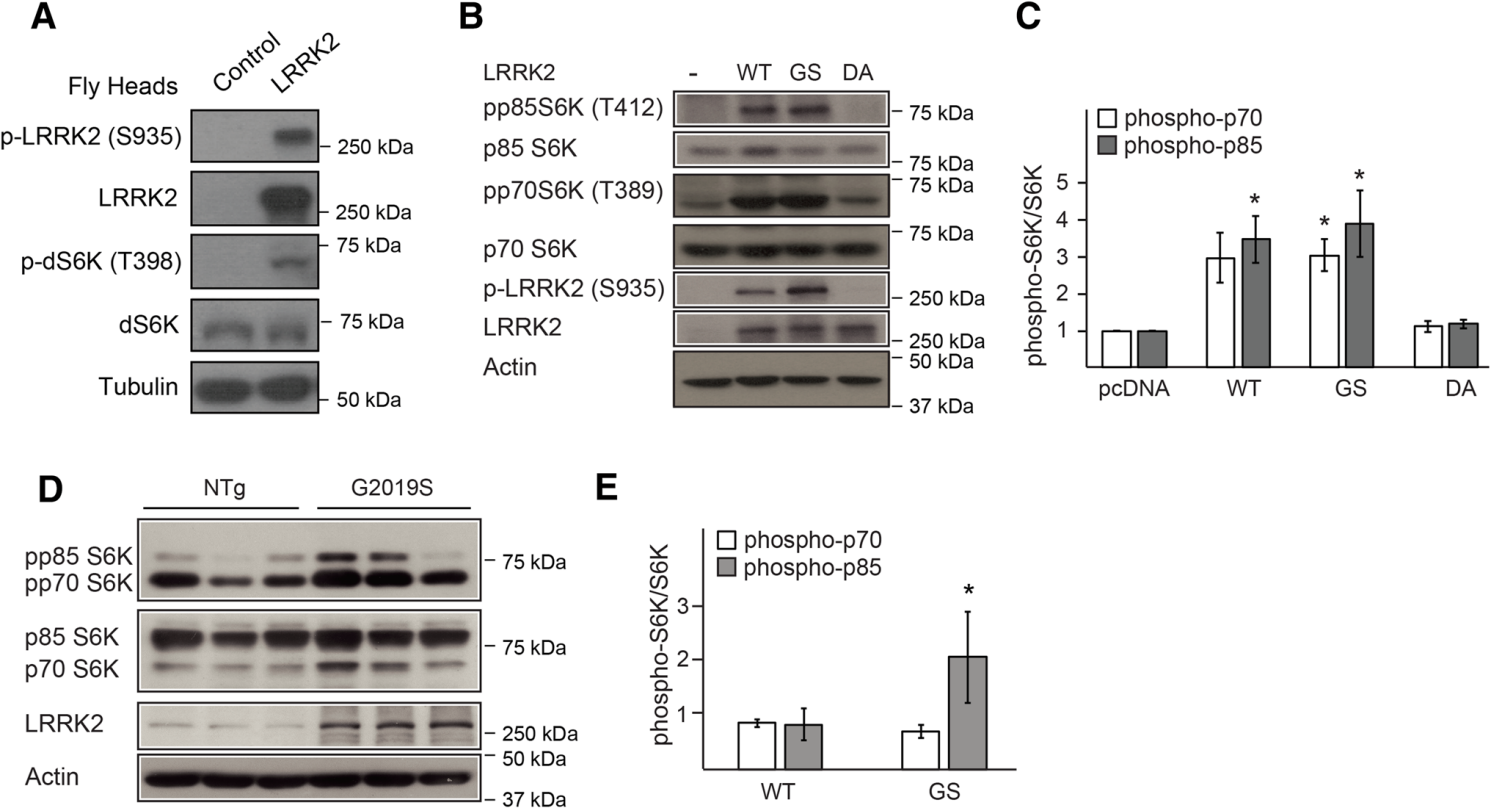
Enhanced phosphorylation of ribosomal S6Kinase in the presence of LRRK2 overexpression **a** Immunoblots showing the phosphorylation levels of S6K (T398), along with LRRK2 phosphorylation (S935) in the brains of control (*yw*) and LRRK2-expressing flies. **b** Immunoblots showing the expression of total and phosphorylated forms of p85-S6K, p70-S6K and LRRK2 in lysates prepared from transfected SH-SY5Y cells, with actin as the loading control. The associated bar-graph showing the average densitometric units of their expression level is shown in **c**. **d** Immnuoblots showing the expression of total and phosphorylated forms of p85-S6K and p70-S6K, as well as LRRK2 in lysates prepared from primary cortical neurons derived from non-transgenic (NTg) control mice or those expressing the LRRK2 G2019S transgene (G2019S) (*n* = 3 each). The associated bar-graph showing the average densitometric units of their expression level is shown in **e**

PP2A is a major phosphatase in eukaryotic cells that regulates many cellular processes. The key towards understanding how PP2A could modify LRRK2-induced neurotoxicity is the elucidation of the common target(s) that the phosphatase and kinase act on. Among the large repertoire of reported substrates for PP2A, we were particularly attracted to one, i.e. the ribosomal S6 kinase (S6K), that was recently elucidated to be a target of PP2A-B’ and thereby its dephosphorylation by the holoenzyme (Hahn et al. 2010). Our interest in S6K was also fuelled by several recent reports that linked LRRK2-induced neurotoxicity to aberrant protein translation (Gehrke et al. 2010; Imai et al. 2008), a pathway that is also promoted by the activation of S6K (Martin et al. 2014a). To address the potential relationship between S6K and LRRK2, we examined the phosphorylation status of S6K in the brains of control and LRRK2-expressing flies and found that *Drosophila* dS6K phosphorylation is enhanced in the presence of LRRK2 expression (Fig. 3a). We also examined S6K phosphorylation in SH-SY5Y cells ectopically expressing wild type or mutant LRRK2 cDNAs. Notably, S6K exists as two isoforms in mammalian cells, i.e. p70 and p85. Interestingly, both isoforms of S6K exhibit enhanced phosphorylation in SH-SY5Y cells expressing wild-type LRRK2 and LRRK2 G2019S (Fig. 3b, c). As expected, the hyperphosphorylation of S6K is not seen in cells expressing a kinase-dead LRRK2 D1994A mutant (Fig. 3b, c). As an extension of this study, we also examined the phosphorylation status of S6K in primary cortical neurons derived from transgenic mice expressing LRRK2 G2019S mutant. Consistent with our observations obtained in SH-SY5Y cells, the phosphorylation of S6K is increased in LRRK2 G2019S-expressing neurons compared to its control counterparts, although it is selective to the p85 isoform (Fig. 3d–e).

### Genetic or Pharmacological Inhibition of S6K Counteracts LRRK2-Induced Neurotoxicity

**Fig. 4:**
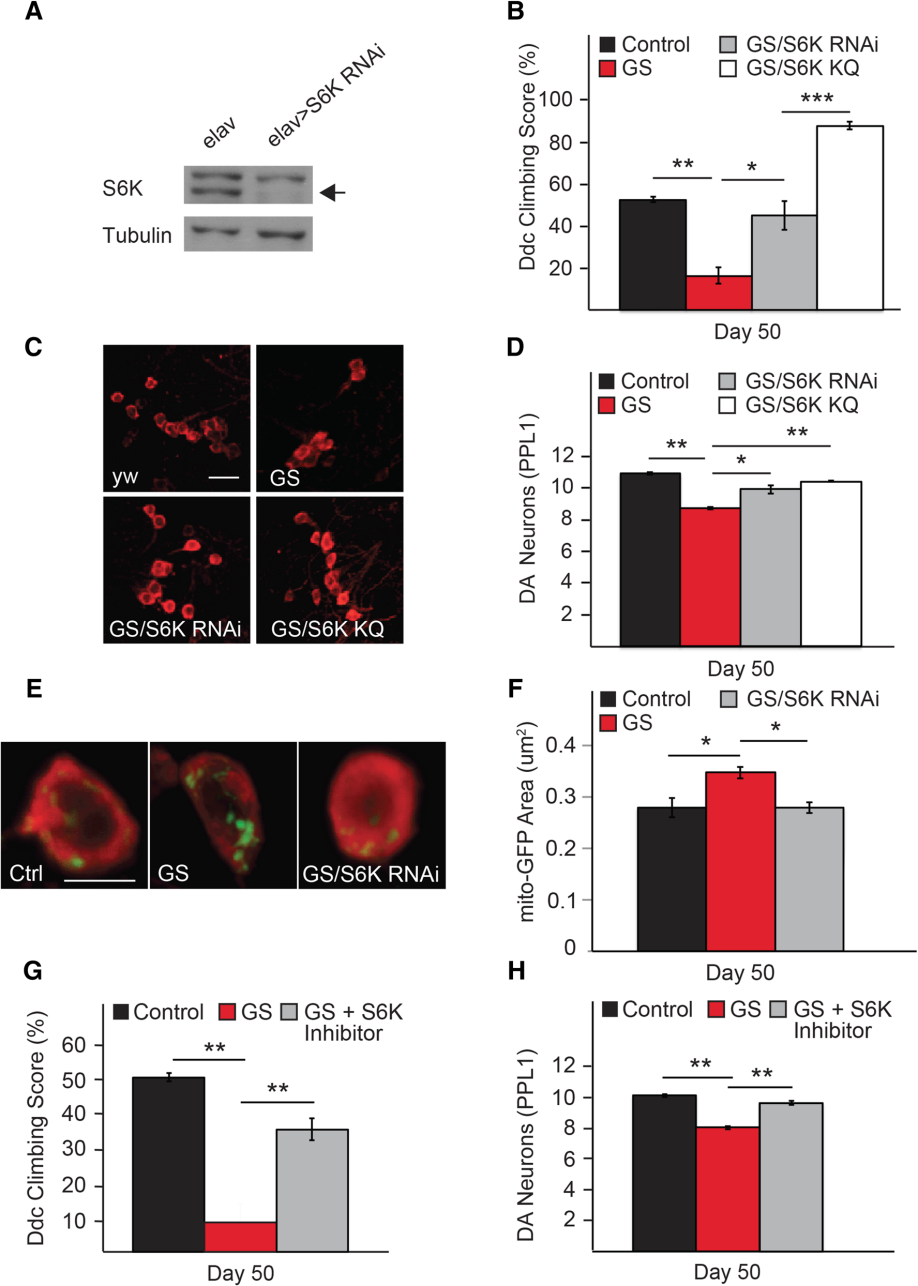
Genetic or pharmacological inhibition of S6K rescues the parkinsonian phenotype of G2019S flies. **a** Immunoblot showing the reduction of S6K expression (indicated by arrow) following UAS-S6K RNAi driven via the *elav*-GAL4 driver. **b** Climbing score of *Ddc*-Gal4 driven LRRK2 G2019S transgenic flies (50 days post-eclosion) in the absence or presence of the co-expression of S6K RNAi or S6K KQ. **c** Representative confocal microscopy images showing TH-positive (red) dopaminergic neurons in the PPL1 cluster of control flies or those expressing LRRK2 G2019S mutant in the absence or presence of Ddc-GAL driven expression of S6K RNAi or S6K KQ dominant negative mutant, as indicated. **d** DA neuronal count of *Ddc*-Gal4 driven LRRK2 G2019S transgenic flies (50 days post-eclosion) in the absence or presence of the co-expression of S6K RNAi or S6K KQ. **e** Representative images of mito-GFP (green) immunostaining in the cell bodies (red) of DA neurons of control flies or those expressing LRRK2 G2019S mutant in the presence or absence of S6K RNAi co-expression. **f** Bar-graph showing the average size ± S.E.M of mito-GFP puncta in control and mutant LRRK2 flies (G2019S) in the absence or presence of S6K RNAi co-expression. **g** Climbing score or **h** DA neuronal count of *Ddc*-Gal4 driven LRRK2 G2019S transgenic flies (50 days post-eclosion) in the absence or presence of S6K inhibitor PF-4708671. *p < 0.05, **p < 0.01

Following the above experiments, we were interested to know if S6K could modulate LRRK2-induced toxicity in vivo. For this purpose, we performed genetic epistasis experiments in LRRK2 expressing flies to examine whether genetic inhibition of S6K via siRNA means or the expression of a dominant negative mutant of S6K (KQ) would mitigate their disease-associated phenotypes. The knockdown of S6K expression via S6K siRNA was first ascertained by expressing the siRNA species in a pan-neuronal manner by means of the elav-GAL4 driver (Fig. 4a). When driven by the *Ddc*-GAL4 driver, we found that both strategies effectively rescue the climbing deficits of the LRRK2 mutant flies (Fig. 4b). No such improvements in climbing performance was recorded when S6K was similarly manipulated in control flies (not shown). At the same time, the expression of S6K siRNA also resulted in a modest albeit significant protection of the DA neuronal number (Fig. 4c, d) that is accompanied by an improved neuronal mitochondrial phenotype (Fig. 4e, f) in LRRK2 mutant flies. Similar outcomes were observed in LRRK2 G2019S-expressing flies expressing the S6K (KQ) mutant (Fig. 4c, d).

As an alternative approach, we also treated LRRK2-mutant flies with PF-4708671, a selective inhibitor of S6K. Consistent with our above genetics-based results, pharmacological inhibition of S6K similarly rescue the climbing and dopaminergic neuronal phenotype of LRRK2 G2019S-expressing flies (Fig. 4g, h) but otherwise has no apparent effects on the climbing performance of control flies (Fig. S1C). Interestingly, in the presence of the S6K inhibitor treatment, expression of *wrd (dPP2A*-*B’)* or *mts* did not promote the climbing performance of LRRK2-mutant flies beyond that brought about by S6K inhibition alone (Fig. S1E), suggesting the attractive possibility that PP2A-mediated protection against LRRK2-induced neurotoxicity likely occurs via counteracting S6K activity.

## Discussion

In this study, we have identified PP2A and S6K as genetic modulators of LRRK2-induced neurotoxicity and demonstrated that the activation of PP2A or inhibition of S6K via genetic or pharmacological means mitigates dopaminergic dysfunction and associated phenotypes in *Drosophila* LRRK2 G2019S mutant. Our results support the suggestion that LRRK2-related phosphatases may be viable therapeutic targets for PD (Taymans 2017).

Interestingly, Lobbestael et al. previously reported that protein phosphatase 1 (PP1) interacts with and dephosphorylates LRRK2 in vitro (Lobbestael et al. 2013). However, we did not identify PP1 as a modulator of LRRK2-induced toxicity in our *Drosophila*-based phosphatase screen. Instead, we have identified the three components of PP2A, i.e., scaffolding (PP2A-29B), regulatory (wdb) and catalytic (mts) subunits, which are required to form a functional holoenzyme, as genetic modulators of LRRK2 function. Our finding is consistent with a recent report by Athanasopoulos and colleagues who found that LRRK2 is able to interact with all the three subunits of PP2A in cultured cells, and that silencing the catalytic subunit of PP2A by shRNA aggravated cellular degeneration induced by the pathogenic LRRK2 R1441C mutant (Athanasopoulos et al. 2016). Here, we have independently verified the finding by means of a different approach using an in vivo model system. Importantly, we have demonstrated the relevance of PP2A in DA neurons expressing the LRRK2 G2019S transgene. Moreover, we showed that treatment of LRRK2-mutant flies with fingolimod, a drug that is currently being used to treat multiple sclerosis, ameliorates their disease-associated phenotypes but otherwise has no apparent effects on the climbing performance of control flies. All the compounds tested also have no overt effects on other DA neuronal cluster examined except for PPL1, which they exert a positive effect. In the presence of PP2A overexpression, we found that LRRK2 phosphorylation at Ser-1292 is reduced, which suggests a mechanism for how PP2A may regulate LRRK2 activity. This is consistent with a recent report demonstrating that Ser1292 dephosphorylation is mediated by phosphatases that are sensitive to calyculin A (PP1) and okadaic acid (PP2) (Reynolds et al. 2014). At the same time, we also found that S6K, a reported PP2A substrate (Bielinski and Mumby 2007; Hahn et al. 2010), functionally interacts with LRRK2. In the presence of LRRK2, S6K phosphorylation is enhanced, although we do not know if their relationship is direct or indirect. Notwithstanding this, given that LRRK2-mediated enhancement of protein translation is thought to underlie its neurotoxicity (Imai et al. 2008; Martin et al. 2014a), it is perhaps not surprising to note that the modulation of S6K activity, which normally promotes protein synthesis via the phosphorylation of ribosomal S6, can influence LRRK2 actions. Notably, the ribosomal protein s15 was recently identified as a key pathogenic substrate of LRRK2 (Martin et al. 2014b). Whether PP2A can reverse the phosphorylation of s15 by LRRK2 is unclear but would undoubtedly be an important question to address. Another area worth investigating is the potential relationship between LRRK2-induced enhancement in protein synthesis and mitochondrial dysfunction, particularly in view of our finding that S6K inhibition can rescue the abnormally enlarged mitochondrial size in DA neurons of LRRK2 mutant flies. It is tempting to speculate that the TOR pathway, which coordinates protein synthesis and mitochondrial activity, may help to connect the dots. Indeed, rapamycin-mediated inhibition of mTOR was reported to rescue the pathological phenotype of LRRK2 flies (Tain et al. 2009). Moreover, Penny and colleagues recently showed that the TOR pathway is involved in the regulation of synaptic homeostasis by LRRK2 in *Drosophila* neuromuscular junction (Penney et al. 2016). Interestingly, the authors further found that LRRK2 collaborates with S6K to promote synaptic enhancement of neurotransmitter release at the neuromuscular junction. It is noteworthy to mention that mTOR-mediated phosphorylation of S6K at Thr-389 is pivotal for its activation (Burnett et al. 1998). This is the same site that we have observed to exhibit enhanced phosphorylation (for p70 S6K) in the presence of LRRK2. Clearly, future studies should be conducted to unravel how the various components linked to LRRK2 pathways converge on impairing protein synthesis and mitochondrial function to bring about pathogenic outcomes.

## Electronic supplementary material

Below is the link to the electronic supplementary material.

Supplementary material 1 (DOCX 815 kb)
