# Supplementary material for: Identification of PP2A and S6 Kinase as Modifiers of Leucine-Rich Repeat Kinase-Induced Neurotoxicity
Source: Neuromolecular Med. 2019 Oct 29;22(2):218–26. doi: 10.1007/s12017-019-08577-z (PMC7230064; doi:10.1007/s12017-019-08577-z)
Supplement: Supplementary file 1 — Supplementary material 1 (DOCX 815 kb) [file 12017_2019_8577_MOESM1_ESM.docx]

**Table S1: List of Phosphatases in *Drosophila*-based RNAi screen**

| **Locus Tag** | **Official Symbol** | **Official Full Name** | **Other Names** | **Hit** |
| --- | --- | --- | --- | --- |
| CG1455 | CanA1 | Calcineurin A1 | PP2B 21EF | - |
| CG1906 | alph | alphabet | SK3-1 | - |
| CG2096 | flw | flapwing | PP1β9C, PP1, PP1c, PP1 9C, PP1β, Pp1β-9C | - |
| CG2984 | Pp2C1 | Protein phosphatase 2C | PP2C, dpp2c1 | - |
| CG3530 | CG3530 | - | Myotubularin-like phosphatase domain; Protein-tyrosine/Dual specificity phosphatase | - |
| CG3632 | CG3632 | - | Myotubularin-like phosphatase domain; Protein-tyrosine/Dual specificity phosphatase | - |
| CG4209 | CanB | Calcineurin B | CNB | - |
| CG4733 | CG4733 | - | PR72, dPR72, PP2A-B'' | - |
| CG5026 | CG5026 | - | Myotubularin-like phosphatase domain; Pleckstrin homology-like domain | - |
| CG5643 | wdb | widerborst | PP2A-B', BcDNA:LD34343, B56-2 | + |
| CG5650 | Pp1-87B | Protein phosphatase 1 at 87B | Su(var)3-6, PP1α87B, PP1, Su-var(3)6, PP1 87B, ck19, PP187B, l(3)j6E7, PP1c, PP1α-87B | - |
| CG7109 | mts | microtubule star | PP2a, PP2Ac, PP2a 28D, PP2A-C, PP2, MTS/PP2A, dPP2A, PP2A C | + |
| CG7115 | CG7115 | - | Protein phosphatase 2C | - |
| CG7134 | cdc14 | cdc14 | - | - |
| CG7180 | Ptp36E | Protein tyrosine phosphatase 36E | DPTP36E | - |
| CG7378 | CG7378 | - | Protein-tyrosine/Dual specificity phosphatase | - |
| CG8402 | PpD3 | Protein phosphatase D3 | PP5 | - |
| CG8822 | PpD6 | Protein phosphatase D6 | - | - |
| CG8980 | NiPp1 | Nuclear inhibitor of Protein phosphatase 1 | NiPp-1, NIPP1Dm | - |
| CG9493 | Pez | Pez | Protein tyrosine phosphatase activity | - |
| CG9819 | CanA-14F | Calcineurin A at 14F | MRE12 | - |
| CG9842 | Pp2B-14D | Protein phosphatase 2B at 14D | canA, PP2B 14D, CnnA14D | - |
| CG10089 | CG10089 | - | Protein-tyrosine/Dual specificity phosphatase | - |
| CG10138 | PpD5 | Protein phosphatase D5 | - | - |
| CG10371 | Plip | PTEN-like phosphatase | - | - |
| CG10376 | CG10376 | - | Protein phosphatase 2C | - |
| CG10493 | Phlpp | PH domain leucine-rich repeat protein phosphatase | Protein phosphatase 2C-like domain | + |
| CG10930 | PpY-55A | Protein phosphatase Y at 55A | PPY, PPY 55A (Calcineurin-like phosphoesterase domain) | + |
| CG11597 | CG11597 | - | Calcineurin-like phosphoesterase domain | - |
| CG12169 | Ppm1 | Ppm1 | - | - |
| CG13197 | CG13197 | - | Protein-tyrosine/Dual specificity phosphatase | - |
| CG14080 | Mkp3 | Mitogen-activated protein kinase phosphatase 3 | DMKP-3 (Protein-tyrosine/Dual specificity phosphatase) | + |
| CG14211 | MKP-4 | MAPK Phosphatase 4 | - | - |
| CG17291 | Pp2A-29B | Protein phosphatase 2A at 29B | Pp2A, CG13383, PP2A 29B, PP2A-A, CG33297 | + |
| CG17598 | CG17598 | - | Protein phosphatase 2C | - |
| CG17746 | CG17746 | - | Protein phosphatase 2C | + |
| CG18339 | Pp4-19C | Protein phosphatase 19C | PP4, PPP4c | - |
| CG31795 | IA-2 | IA-2 ortholog | ia2 (protein tyrosine phosphatase activity) | - |
| CG42327 | CG42327 | - | Protein-tyrosine/Dual specificity phosphatase | - |


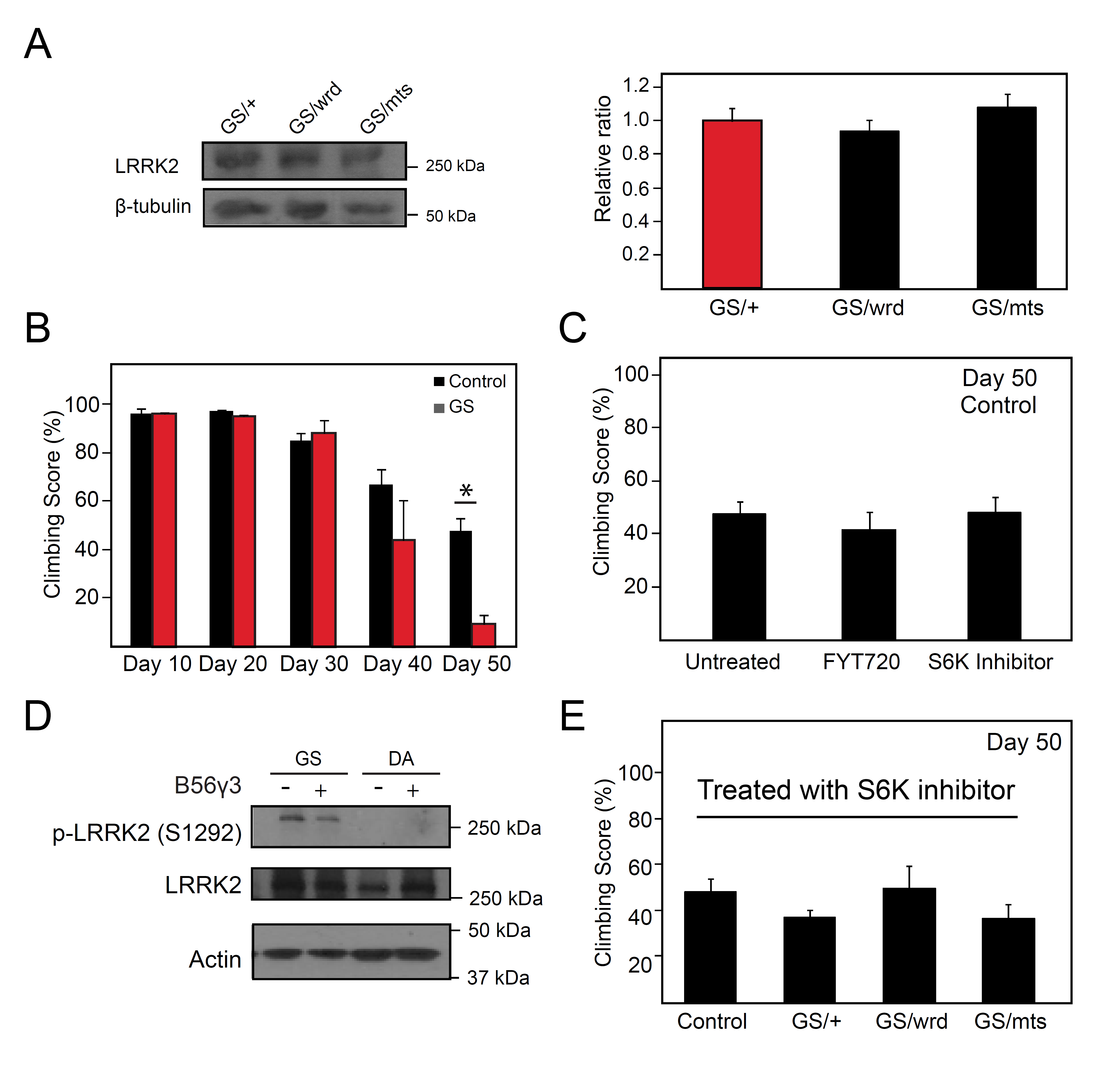


**Figure S1. Characterization of LRRK2 G2019S flies in the presence or absence of PP2A co-expression** (A) Immunoblots showing the expression of LRRK2 in G2019S expressing transgenic flies alone and in the presence of wrd (dPP2A-B’) or mts (dPP2A-C), with β-tubulin as the loading control. The average densitometric unit of their expression levels is shown in the accompanying bar-graph. (B) Climbing score of control (*yw*) and LRRK2 G2019S flies from Day 10 to Day 50 post-eclosion. (C) Climbing score of control (*yw*) flies in the absence or presence of PP2A activator FTY720 or S6K inhibitor PF-4708671 treatment (50 days post-eclosion). (D) Representative immunoblots showing the phosphorylation levels of G2019S and D1994A LRRK2 when co-transfected with PP2A B’ (B56γ3) subunit in SHSY5Y cells. *(p<0.05), **(p<0.01). (E) Climbing score of S6K inhibitor-treated control (*yw*) and LRRK2 G2019S transgenic flies in the absence or presence of wrd (dPP2A-B’) or mts (dPP2A-C) using *Ddc*-Gal driver at day 50 post-eclosion.
